# Value of blended teaching in graduate tobacco medicine training: A prospective intervention study

**DOI:** 10.18332/tid/216380

**Published:** 2026-03-03

**Authors:** Hongjun Wang, Junyan Zhang, Xiaoping Xu, Jia Zhou

**Affiliations:** 1 First Affiliated Hospital of Chongqing Medical University, Chongqing, China

**Keywords:** intervention study, tobacco medicine training, blended teaching, graduate medical students, medical education

## Abstract

**INTRODUCTION:**

Tobacco use remains a critical global public health challenge, particularly in developing countries. As future healthcare providers, medical students and physicians play important roles in smoking cessation interventions. This study evaluates how systematic tobacco medicine training impacts medical graduate students’ smoking cessation knowledge and attitudes.

**METHODS:**

This prospective intervention study was conducted at Chongqing Medical University, China, from 2022 to 2024. A total of 540 graduate students were enrolled and randomly assigned (1:1) to an untrained group (n=270) or a trained group (n=270). The untrained group received no training, while the trained group underwent a 6-week blended tobacco medicine training, comprising 12 online and six offline courses. Post-intervention, an online questionnaire and test were used to assess attitudes and knowledge. Data were analyzed using independent t-tests, Mann-Whitney U tests, chi-squared tests, or Fisher’s exact tests for group comparisons; multivariable linear regression models were employed to adjust for baseline characteristics and evaluate the intervention's impact.

**RESULTS:**

The trained group achieved significantly higher cognitive scores regarding smoking cessation compared to the untrained group (mean difference=1.03; 95% CI: 0.73–1.33, p<0.001). Multivariable regression analysis indicated that training was positively associated with cognitive scores (B=0.92; 95% CI: 0.64–1.20, p<0.001), whereas current smoking status, longer smoking duration, and male gender were negatively associated. Regarding knowledge, the trained group demonstrated higher accuracy rates in most items (p<0.001).

**CONCLUSIONS:**

Systematic blended tobacco medicine training effectively improves medical graduate students’ knowledge and attitudes toward smoking cessation. Integrating such training into medical education is essential for preparing future professionals to support tobacco control.

## INTRODUCTION

Globally, smoking is a rapidly growing public health concern, affecting approximately 1.2 billion adults^1^. Smoking cessation represents the most critical intervention for minimizing smoking-attributable morbidity and mortality^2^. There are more than 300 million current smokers in China. Among these smokers, the smoking cessation rate was only 20.1%, and more than 90% of them had never received any professional help^3^. A nationwide investigation revealed that among China’s approximately 1 million medical and health institutions, only 366 were smoking cessation clinics, indicating a significant shortage in such services, and the majority of them were situated in tertiary hospitals of large cities, which made visiting rather inconvenient^3^.

Medical students and later physicians served as social role models for their patients and played an important role in encouraging smokers to quit. Studies have indicated that smoking cessation advice given by non-smoking physicians is more likely to be accepted by their patients, so physicians should set a good example for their patients^4^. Furthermore, physicians’ advice has been proven to be a powerful motivator for smoking cessation in China, potentially more effective than price increases^5^.

Meanwhile, physicians played a crucial role in tobacco control by offering immediate smoking cessation counseling. Although training physicians on tobacco cessation improves their adherence to smoking treatment guidelines^4^, their counseling performance may ultimately have little impact on the smoking status of their patients^6^. Therefore, targeting medical students – who are ‘soon to be’ doctors – offers a critical window for early intervention. Their smoking behaviors, attitudes, and knowledge of tobacco medicine are essential for their ability to effectively control tobacco use in clinical work in the future^7^. A survey conducted among medical students revealed that over 90% believed that health professionals should undergo specialized training on smoking cessation techniques, but less than 10% stated that they had received any training at medical school^8^. In China, only a handful of medical colleges offered tobacco medicine courses for undergraduates, with merely one class hour, and no tobacco medicine-related courses were provided for postgraduates. Lack of training has been identified as a major barrier preventing doctors from supporting smokers to quit. During the postgraduate period, which is a process from theory to practice, it is crucial for medical postgraduates to acquire effective knowledge of nicotine addiction and tobacco harm, and undergo practical skills training for comprehensive treatment of tobacco dependence. This will enable them to carry out smoking cessation work effectively when they become doctors in the future. Studies conducted in various countries indicate that these issues are insufficiently covered in numerous postgraduate curricula^9,10^, despite evidence suggesting that postgraduate training significantly improves clinical practice in smoking cessation^11^.

Faced with the growing need to adapt to diverse learning environments and overcome barriers in accessibility, many universities have developed online teaching through digital tools such as the Internet, smartphones, and other mobile devices^12^. Many universities have independently established and developed some online teaching platforms, uploading their courses onto the Internet for students to study online, such as the platforms utilized by many Chinese universities like Chaoxing and Rain Classroom. Postgraduate students are required to undertake rotating internships in various departments, thus presenting difficulties in offline learning. Numerous previous studies have compared the effectiveness of offline teaching and online self-study. Some investigations have revealed that self-study is more effective due to its initiative and productivity^13^. However, other studies have indicated that online learning is less effective for certain students lacking independent learning ability due to the lack of constraint and on-site interaction^14^.

This prospective intervention study aims to compare the differences in tobacco control cognition and tobacco-related knowledge between a blended trained group and an untrained group of medical postgraduate students at Chongqing Medical University.

## METHODS

### Study design and participants

This prospective intervention study was conducted at Chongqing Medical University in China from 2022 to 2024. A total of 540 postgraduate medical students were recruited. Inclusion criteria were full-time enrollment in the postgraduate medical program. All participants provided informed consent. The study protocol was approved by the Medical Ethics Committee of The First Affiliated Hospital of Chongqing Medical University (Approval No. 2025-555-01).

### Sample size

The sample size calculation was based on the expected improvement in primary outcome measures, including both knowledge and cognitive scores. Assuming a conservative small-to-medium effect size (Cohen’s d=0.3) to ensure adequate statistical power for all indicators, with a two-sided significance level (α) of 0.05 and a power (1-β) of 80%, the required sample size was calculated to be approximately 176 participants per group. To account for potential attrition and ensure robust multivariable analysis, we expanded the enrollment to include 270 participants per group.

### Randomization and intervention

Participants were allocated in a 1:1 ratio using a computer-generated random number sequence. Those in the Trained group, enrolled in a blended 6-week tobacco medicine training course, which included online learning material, lectures, and seminars on the Chaoxing platform, and offline practical skills training and discussion. Students received 18 tobacco-related instructional modules consisting of 12 online sessions and six offline sessions, each comprising 40 minutes of standard class time. The offline training included scenario simulation, role-playing, and other training on clinical practice related to smoking cessation, including the application of the 5As (Ask, Advise, Assess, Assist, Arrange) brief smoking cessation intervention. Strict attendance records were maintained to ensure adherence. The Untrained group did not receive the specific tobacco-related training during the study period.

### Data collection and outcomes

At the end of the training, an evaluation using a questionnaire created via Tencent Docs and an online exam was conducted to assess and compare the attitudes and knowledge of both groups.

*Demographic information*

We collected demographic information and defined the key variables as follows: age, smoking status (non-smokers; former smokers; and current smokers, smoking at the time of the survey). Specialization was categorized into: Internal medicine, Surgery, and Other (based on the participants’ major field of study).

*Attitudes (cognitive score)*

Evaluated on a scale from ‘strongly agree’ to ‘strongly disagree’ regarding the role of medical students and physicians in tobacco control, attitudes towards smoking among patients, health risks, and efficacy of interventions (Supplementary file Table 1). The questionnaire demonstrated acceptable internal consistency (Cronbach’s alpha=0.70).

*Knowledge*

The assessment included the mechanism and comprehensive treatment of tobacco dependence, health hazards, 5As intervention, electronic cigarettes, and withdrawal symptoms. Scores were calculated based on correct responses (1 point for single-choice, 2 points for multiple-choice).

### Statistical analysis

The analysis included all randomized participants, as every participant completed the study strictly according to the protocol without attrition or deviation. Consequently, the analysis aligns with the intention-to-treat (ITT) principle by preserving the benefit of randomization.

The collected data were organized and analyzed using SPSS version 26 (IBM, Chicago, IL, USA). Categorical variables are presented as frequencies and percentages and were compared using the chi-squared (χ^2^) test or Fisher’s exact test. Continuous variables are presented as mean ± standard deviation (SD) and compared using independent t-tests or rank-sum tests.

To investigate factors influencing cognitive and knowledge scores, univariable and multivariable linear regression analyses were performed. Variables with statistical significance (p<0.05) in the univariable analysis were included in the multivariable models. Multicollinearity was assessed using the variance inflation factor (VIF), and autocorrelation was checked using the Durbin-Watson statistic. Regression coefficients (B) are reported with 95% confidence intervals (CI). A p<0.05 was considered statistically significant.

## RESULTS

### Demographics and baseline characteristics

The study included two groups: a trained group (n=270) and an untrained group (n=270), both achieving a 100% response rate. Participants’ ages ranged from 22 to 29 years. The mean ages were 24.75 ± 1.54 years for the untrained group and 24.99 ± 1.56 years for the trained group (p>0.05). There were no significant differences in gender distribution or smoking behavior between the groups (p>0.05). However, there was a significant difference in specialization distribution (p<0.001), with the trained group having a higher proportion of surgical students (30.7%) compared to the untrained group (14.1%) (Table 1).

**Table 1:** Demographic characteristics and smoking behavior of medical graduate students at Chongqing Medical University, Chongqing, China, 2022–2024 (N=540)

*Characteristics*	*Untrained group n (%)*	*Trained group n (%)*	*t/χ^2^*	*p*
**Age** (years), mean ± SD	24.75 ± 1.54	24.99 ± 1.56	-1.75^a^	0.08
**Gender**			3.7^b^	0.06
Male	101 (37.4)	123 (45.6)		
Female	169 (62.6)	147 (54.4)		
**Smoking status**			2.23^c^	0.35
Non-smoker	255 (94.4)	261 (96.7)		
Former smoker	6 (2.2)	2 (0.7)		
Current smoker	9 (3.3)	7 (2.6)		
**Specialization**			22.78^b^	<0.001
Internal medicine	93 (34.4)	85 (31.5)		
Surgery	38 (14.1)	83 (30.7)		
Other	139 (51.5)	102 (37.8)		

a Independent t-test.

b Chi-squared test.

c Fisher’s exact test.

### Attitudes toward smoking cessation

**Figure 1:**
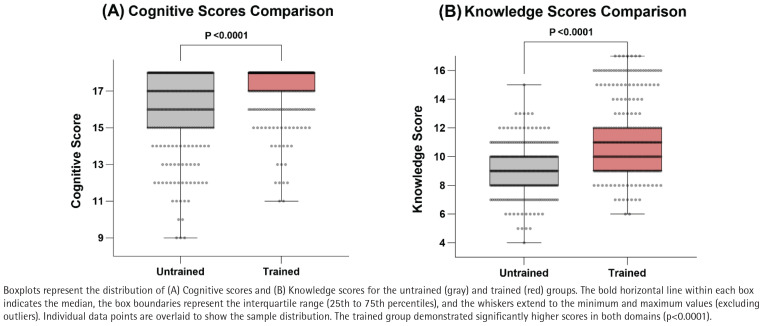
Distribution of cognitive and knowledge scores among medical graduate students at Chongqing Medical University, Chongqing, China, 2022–2024 (N=540)

The results presented in Table 2 and Figure 1A show statistically significant positive differences in attitudes in the trained group compared to the untrained group (p<0.001). For example, 96.2% of the trained group fully agreed that medical students have a duty to remind and help patients quit smoking, compared to 85.6% in the untrained group. Similarly, 82.6% of the trained group agreed that medical students should refrain from smoking to set a positive example, compared to 67.4% in the untrained group.

**Table 2:** Differences in attitudes toward smoking cessation post-intervention among medical graduate students at Chongqing Medical University, Chongqing, China, 2022–2024 (N=540)

*Items*	*Untrained group n (%)*	*Trained group n (%)*	*Z*	*p*
**Medical students have a duty to remind and help patients quit smoking**			-4.34	<0.001
Disagree	13 (4.8)	3 (1.1)		
Partially agree	26 (9.6)	7 (2.6)		
Fully agree	231 (85.6)	260 (96.2)		
**Medical students’ advice is helpful for patients to quit smoking**			-3.76	<0.001
Disagree	7 (2.6)	2 (0.7)		
Partially agree	66 (24.4)	36 (13.3)		
Fully agree	197 (73.0)	232 (85.9)		
**Patients have many health problems to address, so quitting smoking is not that important**			-3.55	<0.001
Fully agree	20 (7.4)	4 (1.5)		
Partially agree	33 (12.2)	21 (7.8)		
Disagree	217 (80.4)	245 (90.7)		
**Encouraging smoking cessation may be less effective and too late, as the damage to patients’ health has become largely irreversible**			-3.71	<0.001
Fully agree	13 (4.8)	7 (2.6)		
Partially agree	39 (14.4)	15 (5.6)		
Disagree	218 (80.7)	248 (91.9)		
**Medical students should refrain from smoking to set a positive example for patients**			-4.13	<0.001
Disagree	14 (5.2)	5 (1.9)		
Partially agree	74 (27.4)	42 (15.6)		
Fully agree	182 (67.4)	223 (82.6)		
**Tobacco consumption is a matter of personal preference, so doctors should not intervene**			-5.8	<0.001
Fully agree	14 (5.2)	9 (3.3)		
Partially agree	107 (39.6)	47 (17.4)		
Disagree	149 (55.2)	214 (79.3)		

Analysis performed using the Mann-Whitney U test.

### Knowledge acquisition

As illustrated in Figure 1B, the overall knowledge scores were significantly higher in the trained group compared to the untrained group (p<0.001). Specifically, the trained group achieved significantly higher correct response rates for most single-choice and multiple-choice questions (Table 3). For instance, in Single-Choice 1 (harms of tobacco), the correct rate was 30.0% in the trained group versus 12.6% in the untrained group (p<0.001). In Multiple-Choice 5 (behavioral replacement therapies), the correct rate was 75.2% versus 59.3% (p<0.001). However, no significant differences were observed for Single-Choice 3 and Multiple-Choice 2 (p>0.05). Notably, for Multiple-Choice 7 (5As intervention), while the trained group performed significantly better (p<0.001), the absolute correct rates were low in both groups (11.5% vs 1.1%).

**Table 3:** Differences in correct response rates for smoking cessation knowledge questions among medical graduate students at Chongqing Medical University, Chongqing, China, 2022–2024 (N=540)

*Questions*	*Untrained n (%)*	*Trained n (%)*	*χ^2^*	*p*
**Single-choice**				
1	34 (12.6)	81 (30.0)	24.41	<0.001
2	190 (70.4)	240 (88.9)	28.54	<0.001
3	263 (97.4)	265 (98.1)	0.34	0.559
**Multiple-choice**				
1	42 (15.6)	91 (33.7)	23.95	<0.001
2	205 (75.9)	218 (80.7)	1.84	0.174
3	7 (2.6)	51 (18.9)	37.4	<0.001
4	221 (81.9)	247 (91.5)	10.83	<0.001
5	160 (59.3)	203 (75.2)	15.54	<0.001
6	77 (28.5)	135 (50.0)	26.12	<0.001
7	3 (1.1)	31 (11.5)	24.61	<0.001

Differences were analyzed using the chi-squared test.

### Factors affecting cognitive scores

Multivariable linear regression analysis (Table 4) revealed that receiving training, smoking status (current smoker), smoking duration, specialization, and gender (male) were significant factors influencing cognitive scores (p<0.05). Receiving tobacco medicine training had a substantial positive association with cognitive scores (B=0.92; p<0.001). Conversely, current smokers (B= -2.12; p=0.016), longer smoking duration (B= -0.40; p=0.049), and male gender (B= -0.52; p=0.001) were negatively associated with cognitive scores.

**Table 4:** Univariable and multivariable linear regression analysis of factors affecting cognitive scores regarding smoking cessation among medical graduate students at Chongqing Medical University, Chongqing, China, 2022–2024 (N=540)

*Variables*	*Univariable analysis*		*Multivariable analysis*				
	*B*	*p*	*B*	*95% CI*	*β*	*p*	*VIF*
**Constant**			16.67	16.39–16.96	-	<0.001	-
**Received training**	1.03	<0.001	0.92	0.64–1.20	0.25	<0.001	1.06
**Smoking status**							
Non-smoker ®							
Former smoker	-1.65	0.007	-0.57	-2.16–1.01	-0.04	0.479	1.97
Current smoker	-3.78	<0.001	-2.12	-3.84 – -0.40	-0.2	0.016	4.57
**Smoking duration** (years)	-0.76	<0.001	-0.4	-0.79 – -0.002	-0.18	0.049	5.92
**Smoking quantity**	-0.3	<0.001	0.04	-0.12–0.19	0.04	0.652	4.67
**Specialization**							
Internal medicine ®							
Surgery	0.12	0.565	0.29	-0.11–0.68	0.06	0.154	1.43
Other	-0.63	<0.001	-0.5	-0.81 – -0.18	-0.13	0.002	1.33
**Gender**							
Female ®							
Male	-0.58	<0.001	-0.52	-0.81 – -0.22	-0.14	0.001	1.14

B: unstandardized coefficient. b: standardized coefficient. VIF: variance inflation factor. While the VIF for smoking duration (5.92) suggests some multicollinearity, the variable was retained to control for the cumulative effect of smoking exposure and its clinical relevance. Durbin-Watson statistic=2.04 (indicating no autocorrelation). Cognitive scores ranged from 9 to 18 points. ® Reference categories.

### Factors affecting knowledge test scores

Multivariable linear regression analysis (Table 5) confirmed that tobacco medicine training was a significant positive predictor of total knowledge test scores (B=1.82; p<0.001) after adjusting for other variables. Additionally, surgical specialization was identified as an independent positive predictor (B=1.85; p<0.001).

**Table 5:** Univariable and multivariable linear regression analysis of factors affecting knowledge scores regarding smoking cessation among medical graduate students at Chongqing Medical University, Chongqing, China, 2022–2024 (N=540)

*Variables*	*Univariable analysis*		*Multivariable analysis*				
	*B*	*p*	*B*	*95% CI*	*β*	*p*	*VIF*
**Constant**			8.92	8.55–9.29	-	<0.001	-
**Received training**	2.17	<0.001	1.82	1.46–2.18	0.37	<0.001	1.05
**Specialization**							
Internal medicine ®							
Surgery	2.27	<0.001	1.85	1.34–2.35	0.31	<0.001	1.43
Other	-0.34	0.128	-0.25	-0.65–0.16	-0.05	0.232	1.31
**Gender**							
Female ®							
Male	0.73	0.001	0.12	-0.26–0.49	0.02	0.544	1.09

B: unstandardized coefficient. β: standardized coefficient. VIF: variance inflation factor. Durbin-Watson statistic=1.43 (indicating no autocorrelation). Knowledge scores ranged from 4 to 17 points. ® Reference categories.

## DISCUSSION

The key finding of this study is that a systematic blended tobacco medicine training program significantly improved both the attitudes and knowledge of medical graduate students regarding smoking cessation. Students who underwent training demonstrated a stronger sense of professional responsibility and a better grasp of cessation interventions compared to their untrained peers.

Our study adopted a blended learning model, integrating online and offline instructional methods. Research has indicated that students who engage in autonomous online learning tend to attain high academic achievements, but offline education offers benefits like interaction and real-time feedback^15^. Our approach combined online theoretical modules with offline practice-based learning, including scenario simulation and role play. The results align with Lauerer et al.^15^ and Ockene et al.^16^, who emphasized the value of practical role-play and structured curricula in developing counseling skills. This approach is further supported by recent findings in Karadoğan et al.^17^, where simulation-based learning was well-received by medical students and effective in improving cessation skills. Furthermore, a systematic review by the Digital Health Education Collaboration confirms that blended education often results in greater improvements in skills and satisfaction compared to digital or traditional learning alone^18^. Similarly, the US Preventive Services Task Force (USPSTF) identifies motivational interviewing and the 5As model as central components of smoking cessation interventions for healthcare professionals^19^, further supporting the inclusion of these strategies in our course.

Specifically, at the cognitive level, medical students who had received training exhibited more positive attitudes toward all smoking cessation-related perspectives. Regarding knowledge acquisition, the training group demonstrated superior accuracy on the majority of questions. However, a notable finding was the persistently low performance on the 5As brief smoking cessation intervention question (Multiple-Choice 7) in both groups, despite the significant improvement in the trained group. This finding is consistent with previous research by Martínez et al.^20^ who reported that while healthcare professionals often perform well in the Ask and Advise components, the implementation of Assist and Arrange remains suboptimal due to the complexity of these steps and perceived barriers. This suggests that mastering the complete 5As framework requires more intensive pedagogical reinforcement than what a standard course might offer. Future curricula should potentially increase the weight of clinical simulations focused specifically on the Assist and Arrange steps to bridge this gap.

We also noted that specialization was an independent predictor of knowledge scores, with surgical students performing better. This confirms the necessity of adjusting for specialization in our multivariable models to isolate the true effect of the training intervention. Additionally, male students and current smokers showed lower cognitive scores, consistent with findings by Salgado et al.^8^, highlighting the role of personal behaviors in shaping attitudes. In the context of China, where male smoking prevalence is significantly higher than female prevalence^21^, these gender-based differences in professional attitudes likely reflect broader sociocultural norms that need to be addressed in medical education.

### Limitations

This study has several limitations. First, it was a single-center study, which may restrict the generalizability of the findings. Second, despite randomization, there was an uneven distribution of specializations. Although we adjusted for known covariates in the multivariable analysis, potential residual confounding from unmeasured factors cannot be ruled out. Third, we relied on self-reported data for smoking status, which carries a risk of information and reporting bias. Finally, the lack of pre-intervention assessments limits the ability to quantify the exact magnitude of individual change. Future research should employ multi-center designs with pre- and post-intervention assessments to better evaluate the sustained impact of such training.

## CONCLUSIONS

Systematic tobacco medicine training among medical graduate students can enhance their cognitive level regarding the hazards of tobacco, reinforce their responsibilities in tobacco control efforts, and significantly promote their mastery of related knowledge. Integrating tobacco medicine training into medical curricula is essential to help medical students provide professional smoking cessation advice to patients in the future.

## Supplementary Material



## Data Availability

The data supporting this research are available from the authors on reasonable request.
