# Supplementary material for: Value of blended teaching in graduate tobacco medicine training: A prospective intervention study
Source: Tob Induc Dis. 2026 Mar 3;24:10.18332/tid/216380. doi: 10.18332/tid/216380 (PMC12964558; doi:10.18332/tid/216380)
Supplement: Supplementary file 1 [file TID-24-30-s1.pdf]

Supplementary Table 1. Cognitive scoring table

Opinion	Cognitive Score
**Medical students have a duty to remind and help patients quit smoking.**	
Disagree	1
Partially Agree	2
Fully Agree	3
**Medical students' advice is helpful for patients to quit smoking.**	
Disagree	1
Partially Agree	2
Fully Agree	3
**Patients have many health problems to address, so quitting smoking is not that important.**	
Fully Agree	3
Partially Agree	2
Disagree	1
**Encouraging smoking cessation may be less effective and too late, as the damage to patients' health has become largely irreversible.**	
Fully Agree	3
Partially Agree	2
Disagree	1
**Medical students should refrain from smoking to set a positive example for patients.**	
Disagree	1
Partially Agree	2
Fully Agree	3
**Tobacco consumption is a matter of personal preference, so doctors should not intervene.**	
Fully Agree	3
Partially Agree	2
Disagree	1

Note: Higher scores indicate more accurate cognition.

Supplementary Table 2. Single-Choice questions and options

**1. Which of the following statements about smoking is incorrect?**
A. The amount and duration of smoking are proportional to the risk of developing diseases.
B. Quitting smoking is the most fundamental way to reduce the risk of smoking-related diseases and improve prognosis.
C. Tobacco and smoke contain many harmful chemicals, such as tar, benzopyrene, and dimethylnitrosamine, which are carcinogens.

Supplementary Table 2 (continued)

D. Secondhand smoke includes mainstream smoke exhaled by smokers and sidestream smoke from burning tobacco products, and mainstream smoke is more toxic.
E. There is no significant difference between the harm caused by passive and active smoking.
**2. Which neurotransmitter is associated with the reward and withdrawal processes in smoking cessation?**
A. Dopamine
B. Acetone
C. Acetylcholine
D. Serine
**3. Is tobacco dependence a chronic addictive disease?**
A. Yes
B. No

Supplementary Table 3. Multiple-Choice questions and options

**1. Which of the following statements about the relationship between smoking and diseases are correct?**
A. Long-term exposure to secondhand smoke damages airway epithelium and increases the risk of respiratory diseases, but short-term exposure has minimal effects.
B. Smoking is closely associated with the incidence and mortality of multiple diseases, with the top three smoking-related causes of death being malignant tumors, cardiovascular diseases, and respiratory diseases.
C. Smoking contributes to the development and progression of atherosclerosis (AS), increases the risk of acute myocardial infarction (AMI), and raises the risk of stroke.
D. Smoking can cause insulin resistance, leading to diabetes.
E. Smoking causes premature skin aging and is significantly linked to baldness.
**2. What are the effects of smoking on the respiratory defense mechanism?**
A. Smoking impairs the mucociliary transport system in the respiratory tract.
B. Smoking increases the sensitivity of the cough reflex.
C. Smoking compromises the immune system.
D. Smoking reduces macrophage function.
**3. Which of the following are common misconceptions about smoking cessation?**
A. Sudden smoking cessation after long-term smoking can cause diseases such as lung cancer.
B. Quitting smoking causes weight gain, so “smoking for weight control” is advisable.

Supplementary Table 3 (continued)

C. Eliminating indoor smoking is the only scientifically proven way to address secondhand smoke.
D. Purchasing cigarettes labeled “low tar” reduces health risks.
E. Choosing filtered, high-quality cigarettes minimizes harm.
**4. What are common symptoms during smoking cessation?**
A. Nicotine craving
B. Irritability and mood swings<br>C. Reduced attention and concentration<br>D. Sleep problems
E. Headache and dizziness
**5. Which behavioral replacement therapies help alleviate nicotine cravings during smoking cessation?**
A. Reading newspapers
B. Drinking milk
C. Consuming snacks
D. Finger massage
E. Moderate exercise
**6. Which of the following are recognized pharmacological treatments for smoking cessation?**
A. Nicotine gum
B. Nicotine patches
C. Varenicline
D. Sustained-release bupropion hydrochloride
E. Using electronic cigarettes
**7. What are the components of the “5A” brief smoking cessation intervention?**
A. Ask: Inquire if the patient smokes and record it.
B. Advice: Recommend that all smokers quit.
C. Assess: Evaluate the smoker’s willingness to quit.
D. Achieve: Help smokers achieve cessation.
E. Acknowledge: Let the patient recognize the harm of smoking.

© 2026 Wang H. et al.
